# A risk stratification tool for hospitalisation in Australia using primary care data

**DOI:** 10.1038/s41598-019-41383-y

**Published:** 2019-03-21

**Authors:** Sankalp Khanna, David A. Rolls, Justin Boyle, Yang Xie, Rajiv Jayasena, Marienne Hibbert, Michael Georgeff

**Affiliations:** 1 CSIRO Australian e-Health Research Centre, Brisbane, QLD 4029 Australia; 2 CSIRO Australian e-Health Research Centre, Melbourne, VIC 3052 Australia; 3 CSIRO Australian e-Health Research Centre, Sydney, NSW 2122 Australia; 4 Precedence Health Care, Melbourne, VIC 3000 Australia

## Abstract

Predictive risk models using general practice (GP) data to predict the risk of hospitalisation have the potential to identify patients for targeted care. Effective use can help deliver significant reductions in the incidence of hospitalisation, particularly for patients with chronic conditions, the highest consumers of hospital resources. There are currently no published validated risk models for the Australian context using GP data to predict hospitalisation. In addition, published models for other contexts typically rely on a patient’s history of prior hospitalisations, a field not commonly available in GP information systems, as a predictor. We present a predictive risk model developed for use by GPs to assist in targeting coordinated healthcare to patients most in need. The algorithm was developed and validated using a retrospective primary care cohort, linked to records of hospitalisation in Victoria, Australia, to predict the risk of hospitalisation within one year. Predictors employed include demographics, prescription history, pathology results and disease diagnoses. Prior hospitalisation information was not employed as a predictor. Our model shows good performance and has been implemented within primary care practices participating in Health Care Homes, an Australian Government initiative being trialled for providing ongoing comprehensive care for patients with chronic and complex conditions.

## Introduction

The growing burden of chronic conditions (also known as non-communicable diseases), is now responsible for 70% of deaths globally^1^. Chronic conditions affect one in two people in developed countries and the World Health Organisation attributes three quarters of global chronic condition-related deaths to developing countries^1^. There is a strong imperative to address this disproportionate burden of chronic conditions on health care globally.

Worldwide efforts to move from episodic to integrated and coordinated care have delivered significant improvements in the management of chronic conditions in the primary care sector, demonstrating benefits to patients and the healthcare system^2–7^. To ensure optimal use of limited healthcare resources, it is crucial that such programs and resources target patients who would otherwise be admitted for an unplanned or avoidable hospitalisation.

The best performing risk stratification algorithms for primary care settings have generally been developed in locations where large linked datasets are practical and available. These datasets can offer good coverage of data variables and include information from outside the primary care setting, such as the patient’s history of previous hospitalisations, a highly significant predictor in such models^8,9^.

However, such linked data sets are not available in most health systems throughout the world. For example, in Australia there are no simple mechanisms to link patient records across primary and acute care settings. Furthermore, patient information about previous hospitalisations is not available in most primary care information systems. In such cases, there is a need for new algorithms based on data from the setting in which they will be used^8^.

Health Care Homes is a new Australian Government initiative with ongoing comprehensive care provided for up to 65,000 patients with chronic and complex conditions in up to 200 primary care clinics and Aboriginal Community Controlled Health Services (their ‘Health Care Home’). Patients eligible for the 22-month trial are identified in the general practice (GP) using an algorithm that predicts the risk of a patient being hospitalised over the next 12 months. By targeting services to patients with an imminent risk of hospitalisation, a significant reduction in the burden on the acute care health system is expected while ensuring valuable resources are allocated to programs and patients in greatest need.

This study describes the development and validation of a prediction model using Australian hospital and GP data to identify patients at risk of hospitalisation for the Health Care Homes trial. The developed prediction model employs routinely collected data from primary care clinics. We assume patient history of hospitalisation is not available as a predictor as this is typically not available in GP clinic databases. The algorithm developed and validated in this study is designed for use in Australian primary care. However, it also illustrates how risk stratification algorithms can be developed and validated to perform adequately in settings where hospitalisation history linked to acute care data may not be readily available.

## Methods

### Study Design and Data Sources

The work described here uses a retrospective cohort comprised of primary care patients whose data is linked with records of hospitalisations. Primary care data for the calendar years 2007 to 2016 was obtained from 29 general practice medical centres in Victoria, Australia. Hospitalisation data for patients admitted to public hospitals during the calendar years 2012 to 2016 in Victoria was provided by the Department of Health and Human Services (DHHS) Victoria.

Ethics approval for this study was provided by the CSIRO Health and Medical Human Research Ethics Committee (CHM HREC) Low Risk Review Panel – (Proposal LR 7/2017). All methods were performed in accordance with the guidelines and regulations relevant to the ethics approval. The ethics committee approved a waiver of consent for several factors including the nature of study, the size of the dataset, the fact that the data had been previously collected, and because the research posed minimum intrusion on people’s privacy.

Both data extracts were de-identified by their source organisation by attaching a unique identifier created using a SHA-256 hash. This helped ensure privacy as no primary identifiers left each data custodian’s organisation. The hashing algorithm used name, date of birth and Medicare number (a unique identifier for patients under the publicly funded universal health care system in Australia). Consistent pre-processing of patient names was undertaken by the custodians of both the primary care and hospitalisation data to ensure a high match rate (e.g. removing punctuation, numbers and spaces from name fields). These datasets were then provided for the study and the SHA field was used to link the primary care and hospitalisation records.

### Participants

The Victorian primary care patient database contained data for over 1.8 million patients. This was filtered to consider only patients who attended their primary care clinics at least once between 2011 and 2016, resulting in approximately 1.3 million patients. To ensure every patient in the resulting cohort had at least one year of history (“look back”) available, and that every patient visited their GP after the one-year prediction period, and was alive for the entire prediction period, two key dates were calculated for each patient. “One year ahead” is one year from the date of a patient’s first visit (minimum value 1 Jan 2012). “One year back” is one year before the date of a patient’s last visit (maximum value 31 December 2015). The patient cohort was subsequently filtered to include only patients where the “One year back” date occurred on or after the “One year ahead” date.

**Figure 1:**
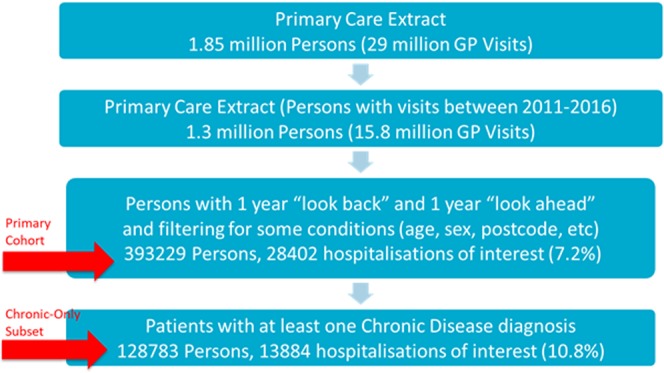
Schematic representing cohort participants at each stage of cohort selection. Note: hospitalisations of interest are Emergency or Potentially Preventable Hospitalisation within 365 days. Chronic-Only subset of patients is a subset of the Primary Cohort that had a Chronic Disease diagnosis (i.e. a diagnosis belonging to any one of the 33 of 35 identified disease families of interest).

The cohort was further filtered to exclude patients below the age of 18 and above the age of 106 years, patients without Victorian postcodes, patients with gender other than “Male” or “Female”, and physiological observations and pathology records with inconsistent or incorrect units and values. Figure 1 presents the cohort population at each stage of the cohort selection process. The resulting cohort, hereafter referred to as the Primary Cohort, comprised 393,229 patients, of whom 7.2% had hospitalisations of interest. A chronic-only subset of the Primary Cohort was also created for sub-analysis and is described in following sections.

**Figure 2:**
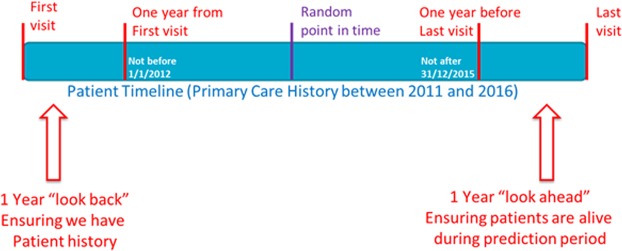
Illustration of Prediction Date as a random day between the “one year ahead” and “one year back” dates. Note: The “look back” and “look ahead” periods ensured that sufficient history and prediction window is available. A random date helped avoid bias while also suiting the perceived consumer use of the model.

To avoid a bias arising from consistently choosing the earliest or latest possible date as a patient’s “Prediction Date”, a random date was chosen between the “One Year Ahead” and “One Year Back” dates. While ensuring that sufficient history and prediction windows were available, this suited the perceived consumer use of the model, where GP practices may want to predict on their cohort at an “ad hoc” time period, not linked to a particular prediction date. Figure 2 presents the methodology chosen for selecting a random prediction date for each patient.

The public hospitalisation extract revealed several patients that appeared to be different people in the primary care dataset but were determined to be the same person. Where this was identified, their primary care records were merged to improve data quality.

### Emergency or Potentially Preventable Hospitalisations

The outcome variable of interest for study was whether a patient has an Emergency or Potentially Preventable Hospitalisation within one year of the prediction date. Potentially Preventable Hospitalisations were included as they are a defined indicator for ambulatory care sensitive conditions^10^ and the case for targeting these through general practice integrated care interventions is well justified^11,12^. An Admission Type flag was employed to identify *Emergency Hospitalisation*. International Classification of Diseases (ICD) and Procedure Code information was employed to identify *Potentially Preventable Hospitalisations* using the guideline provided by the Australian Institute for Health and Welfare^13^. A binary (1/0) “One Year Emergency or Potentially Preventable Hospitalisation” variable was created after calculating, for each patient, the days from their prediction date to their next Emergency or Potentially Preventable Hospitalisation, whichever came first. The data comprised an extract from the inpatient (VAED) and emergency department (VEMD) datasets linked to the death registry (Registry of Births, Deaths and Marriages, Victoria). The only records of interest were related to inpatient hospitalisation.

### Predictors

The list of all predictors used to develop the model is shown in Table 1 with further description below.

**Table 1:** List of available and derived predictors used for model development.

Category	Predictor	Definition	Category	Predictor	Definition
**Demographics**			**Diagnosis Families and Counts**		
	Age	Age at prediction date		Asthma	0, 1; See Supplementary Table S2
	Age_sqrt	sqrt(Age)		COPD	
	Age squared	Age^2^		Chronic kidney disease	
	Age cubed	Age^3^		Coronary heart disease	
	Gender	Male, Female		Stroke	
	Ethnicity	Non-Indigenous Australian, Indigenous Australian, Unknown		Transient Ischemic Attack (TIA)	
	IRSAD (11 categories)	1–10,Unknown		Atrial fibrillation	
	Modified Monash Model	1, 2, 3, 4, 5+, Unknown		Congestive heart failure	
**Patient Observations and History**				Diabetes (type 1)	
	BMI (4 categories)	<25; [25, 30); 30+; Not recorded		Diabetes (type 2)	
	BMI (6 categories)	<25; [25, 30); [30, 35); [35, 40); 40+; Not recorded		Venous thromboembolism	
	Smoking Status	Non-smoker, Ex-smoker, Smoker, Unknown		Osteoarthritis	
	Alcohol Per Day (3 category)	0, 1+, Not recorded		Depression	
	Alcohol Per Day (6 category)	0, 1, 2, 3, 4 +, Not recorded		Anxiety	
	Alcohol Days Per Week	0, 1–2, 3+, Not recorded		Bipolar	
	Any Alcohol Consumption	Non-drinker, Drinker, Not recorded		Schizophrenia	
**Medication Families and Counts**				Cancer	
	Num. prescribed families	0, 1, 2, 3, 4, 5, 6		Dementia	
	Num. prescribed families (5 category)	0, 1, 2, 3, 4+		Learning difficulties	
	Statins	0, 1; See Supplementary Table S1		Falls	
	AntiCoagulants			Epilepsy	
	AntiDepressants			Crohns disease	
	AntiPsychotics			Ulcerative colitis	
	AntiInflammatory			Coeliac disease	
	Steroids			Steatorrhea	
**Pathology Test Results (Missing included)**				Malabsorption syndrome	
	Haemoglobin	Low, Med or High; See Supplementary Table S3		Chronic liver disease	
	Platelets			Pancreatitis	
	Alanine aminotransferase level (ALT)			Hypertension	
	Gamma-glutamyl transferase (GGT)			Osteoporosis	
	Haemoglobin A1c level (HbA1c)			Rheumatoid Arthritis	
	Cholesterol			Hyperlipidaemia	
	Albumin/creatinine ratio (ACR)			Hypercholesterolaemia	
	LDL cholesterol			Hypertriglyceridaemia	
	Estimated glomerular filtration rate (eGFR)			Rheumatic heart disease	
	Blood Pressure			Num. Diagnosis Families	0–35; Num. non-zero diagnosis variables
	Bilirubin			Num. Diagnosis Families squared	(Num Diagnosis Families)^2^
	Creatinine			Num. Diagnosis Families cubed	(Num Diagnosis Families)^3^
	Triglycerides (TAG)			Num. Diagnosis Families (9 categories)	0, 1, 2, 3, 4, 5, 6, 7, 8+
**Pathology Test Results (Missing excluded)**			**Diagnosis Groups**		
	Haemoglobin	Low, Med or High; See Supplementary Table S3		Respiratory	0, 1; See Supplementary Table S2
	Platelets			Atrial fibrillation	
	Alanine aminotransferase level (ALT)			Cardiovascular	
				Osteoarthritis	
	Gamma-glutamyl transferase (GGT)			Osteoporosis	
				Rheumatoid arthritis	
	Haemoglobin A1c level (HbA1c)			Mental health	
	Cholesterol			Cancer	
	Albumin/creatinine ratio (ACR)			Digestive diseases	
	LDL cholesterol			Hypertension	
	Estimated glomerular filtration rate (eGFR)			Bloodfats	
				Chronic kidney disease	
	Blood Pressure			Diabetes (type 1)	
	Bilirubin			Diabetes (type 2)	
	Creatinine			Venous thromboembolism	
	Triglycerides (TAG)			Other Conditions	

#### Demographics

Age at cohort entry was expected to be a key predictor of risk and was implemented as a continuous variable instead of categorical to prevent loss of information within categories. To capture a non-linear relationship of risk on age, the square root of age (√age), age^2^ (age × age) and age^3^ (age × age × age) predictors were also created. This was preferred over Cubic Splines, given that these were more practical to implement within the production environment of software systems used in primary care practices participating in the Health Care Homes trial.

For the Australian context, ethnicity was defined as Indigenous Australians, non-Indigenous Australians, or Unknown (where no ethnicity data was available).

Postcode was used to match with the Australia Bureau of Statistics’ SEIFA Index of Relative Socio-economic Advantage and Disadvantage (IRSAD)^14^, and with the Australian Department of Health’s Modified Monash Model (MMM)^15^. The SEIFA index summarises information about the economic and social conditions of people and households within an area, including both relative advantage and disadvantage measures, and was employed as a categorical decile value (i.e. grouped into 10 bands with lower values signifying higher levels of disadvantage, plus “Unknown” for missing postcodes). MMM is a recently developed geographical classification system designed to better address the maldistribution of medical services across Australia, taking values ranging from 1 to 7, with increasing values representing higher levels of rurality, plus “Unknown” for missing postcodes.

#### Patient Observations and History

Recorded physiological observations were included following data preparation to remove non-numeric characters, conversion of units to standardised forms, and removal of values and units identified as being “incorrectly recorded”. Since all physiological observations are not repeated at each GP visit, the system looked back through all available physiological observations (going back to 2007) to retrieve the most recent available reading.

Smoking status was included as a four-category predictor. Four variations of alcohol consumption were included. In the Australian context, the main software systems collect the number of alcoholic drinks per day consumed by the patient on average, the number of days per week the patient consumed alcohol on average, both of these, or a related measure. To increase the usability of the model in the Australian context, we defined alcohol consumption as a binary condition indicated by any consumption of alcohol per day and alcohol days per week data. We note that “volume” information is lost when converting to a binary category which is commented on in the Discussion. An additional category was included to indicate no alcohol consumption data was available.

Height, weight and body mass index (BMI) were also employed for prediction. BMI was calculated where it was not specifically recorded, but where height and weight were available. Height and weight were used as continuous predictors while BMI was employed as a categorical predictor, either with four categories or with six categories. In the 4-category predictor, the categories corresponded to: underweight/normal, overweight, obese, and “not recorded”. In the 6-category predictor, the obese category was subdivided into moderately obese, severely obese, and very severely obese.

#### Medication Variables

The usage of certain medications as indicated in the GP prescription data was included in the modelling using 0/1 binary indicator variables for each of six medication groups (statins, anticoagulants, antidepressants, antipsychotics, anti-inflammatories, and steroids). Groupings of medications into each medication group was completed in consultation with clinical and pharmacy experts from the Australian Government Department of Health. Data was searched back to 2007 (see Supplementary Table S1). The number of prescribed medication groups was also included in modelling either as the total number of prescribed groups (range 0–6) or as a categorical variable with five categories.

#### Diagnosis Variables

Grouping of diagnosis conditions/diseases of interest into “diagnosis families” was completed in consultation with the Australian Government Department of Health. In total, 35 diagnosis families were identified as relevant for the model (e.g., type 1 and 2 diabetes, osteoporosis, rheumatoid arthritis, cancer, learning difficulties, falls etc.). Data was searched back to 2007. Due to sparsity in the hospitalisation data, these “diagnosis families” were then further combined into 16 “disease groups”. The grouping was logical and clinically relevant (e.g., hyperlipidaemia, hypercholesterolaemia, and hypertriglyceridaemia were combined into a “bloodfats” category), and was informed by the incidence of each of the diagnosis conditions to ensure counts of the employed categorical variable were sufficient for analysis. A 0/1 binary indicator predictor for each group indicated the presence of at least one of the constituent diagnoses of interest in the GP records. The mapping of diagnosed conditions into diagnosis families and diagnosis groups is presented in Supplementary Table S2. These diagnosis group variables were the primary means to include the occurrence of relevant diagnosed conditions/diseases in the models. Note that these predictors correspond to diagnosis, not occurrence; they provide no way to capture additional risk from undiagnosed conditions/diseases of interest. However, the morbidity risk groups from pathology test results do provide a limited way to do this. Comorbidity associated with each patient was also included in modelling as the total number of distinct diagnosis families, either as a continuous predictor (range 0–35) or as a categorical variable with nine categories.

A “Chronic-only” group of diagnosis families was also defined, being one of 33 chronic conditions (i.e. any of the 35, excluding learning difficulties and falls). A “Chronic-only subset” of the patient cohort was then defined as including only those patients who had at least one diagnosis belonging to one of the “Chronic-only” group of diagnosis families.

#### Pathology Variables

Twelve pathology results were included as predictors. Following removal of values and units identified as being “incorrectly recorded”, the most current value of the available pathology results was used. Normal ranges for pathology results were defined in consultation with the Australian Government Department of Health and used to calculate morbidity risk flags representing three categories of risk – Low, Medium and High (see Supplementary Table S3). While not strictly a pathology test result, blood pressure was handled in a similar way. Due to data sparsity, medium and high levels were each combined for Bilirubin, Creatinine and Triglycerides.

For a number of patients, no test results were available. If these were treated as missing values it would be very difficult to include those patients in models using test results as predictors. An alternative approach we employed was to add a category for “no test history” for each predictor which enabled inclusion of all patients. Note that the absence of a test result is not the same as a test result indicating low risk.

### Methodology

A predictive model for the binary event of Emergency or Potentially Preventable hospitalisation within one year was developed. Several machine learning approaches were chosen for model development and validation. Logistic regression^16^ was chosen because it is an established method for prediction in binary problems and implementation of models in a range of production environments is straightforward using basic mathematical functions. For comparison, four alternative types of models were also considered (Naïve Bayes^17^, Artificial Neural Networks^18^, Random Forests^19^ and Generalised Boosting^20^). Random Forests, Artificial Neural Networks and Generalised Boosting algorithms were considered because of their established superiority in pattern recognition from large complex data. Naive Bayes was employed as it offers a different approach to model building. Multiple variants of the full model were explored. For example, BMI with four or six categories, alcohol consumption as drinks per day or non-drinker/drinker, and the number of diseases as a numeric or a categorical variable were each considered. For each model variant, the performance of the logistic regression model and the four machine learning approaches were assessed.

Models were compared and validated using the area under the receiver operating characteristic curve (AUC) with 10-fold cross-validation. Optimism was estimated using bootstrap validatation^16^ with 100 bootstrap samples. For additional results (e.g., receiver operating characteristic (ROC) curves, calibration curves), patients were randomly split into 70%/30% training and test portions.

The subgroup of patients with at least one chronic condition (Chronic-only subset) is an important subgroup of primary care patients, especially since many programs like Health Care Homes tend to focus on patients with established chronic conditions. Although models were developed using the entire cohort of patients, additional results for this subset of patients were obtained and are reported in the Supplementary material (see Supplementary Results for the Chronic-Only Subset). This approach was used instead of restricting all model development and validation to the chronic-only subset to take advantage of the information and large patient numbers amongst those without a diagnosed chronic condition.

### Software

All analyses were performed using the R statistical computing environment. Logistic regression models used the “glm” command. Random forest models employed the “randomforest” package. Generalised boosting models used the “GBM” package. Naïve Bayes models employed the “klaR” package. Artificial Neural Networks models employed the “nnet” package.

### Code Availabilty

The code used for model development and validation is available from the corresponding author on reasonable request for non-commercial purposes.

## Results

### Participants

Table 2 summarises the patient characteristics in the Primary Cohort all together, as split by the outcome variable, and as split randomly into 70% training (development) and 30% testing (validation) datasets. For categorical quantities, values used as the reference category in modelling are listed first.

**Table 2:** Patient Characteristics by Hospitalisation Outcome and by Development/Validation Subsets.

Characteristic	All Patients (n = 393229)	Hospitalised (n = 28402)	Not Hospitalised (n = 364827)	Training (70%) (N = 275259)	Testing (30%) (N = 117970)
**Demographics**					
**Median Age (IQR)**	36 (23)	41 (30)	36 (22)	36 (23)	36 (23)
**Gender, n (%)**					
Male	175472 (44.6)	11683 (41.1)	163789 (44.9)	122830 (44.6)	52642 (44.6)
Female	217757 (55.4)	16719 (58.9)	201038 (55.1)	152429 (55.4)	65328 (55.4)
**Ethnicity, n (%)**					
Non-Indigenous Australian	306345 (77.9)	21985 (77.4)	284360 (77.9)	214340 (77.9)	92005 (78)
Indigenous Australian	1723 (0.4)	205 (0.7)	1518 (0.4)	1216 (0.4)	507 (0.4)
Unknown	85161 (21.7)	6212 (21.9)	78949 (21.6)	59703 (21.7)	25458 (21.6)
**SEIFA IRSAD, n (%)**					
5	51122 (13)	4317 (15.2)	46805 (12.8)	35748 (13)	15374 (13)
1	34527 (8.8)	3549 (12.5)	30978 (8.5)	24134 (8.8)	10393 (8.8)
2	4467 (1.1)	343 (1.2)	4124 (1.1)	3123 (1.1)	1344 (1.1)
3	14081 (3.6)	1357 (4.8)	12724 (3.5)	9916 (3.6)	4165 (3.5)
4	44804 (11.4)	3325 (11.7)	41479 (11.4)	31240 (11.3)	13564 (11.5)
6	42649 (10.8)	2729 (9.6)	39920 (10.9)	30018 (10.9)	12631 (10.7)
7	48160 (12.2)	3040 (10.7)	45120 (12.4)	33708 (12.2)	14452 (12.3)
8	33561 (8.5)	2067 (7.3)	31494 (8.6)	23502 (8.5)	10059 (8.5)
9	78010 (19.8)	5368 (18.9)	72642 (19.9)	54584 (19.8)	23426 (19.9)
10	41090 (10.4)	2248 (7.9)	38842 (10.6)	28771 (10.5)	12319 (10.4)
Unknown	758 (0.2)	59 (0.2)	699 (0.2)	515 (0.2)	243 (0.2)
**Patient Observations**					
**BMI, n (%)**					
<25	44391 (11.3)	2794 (9.8)	41597 (11.4)	31092 (11.3)	13299 (11.3)
[25, 30)	38702 (9.8)	2760 (9.7)	35942 (9.9)	27084 (9.8)	11618 (9.8)
[30, 35)	21581 (5.5)	1939 (6.8)	19642 (5.4)	15130 (5.5)	6451 (5.5)
[35, 40)	8940 (2.3)	902 (3.2)	8038 (2.2)	6303 (2.3)	2637 (2.2)
40+	5949 (1.5)	727 (2.6)	5222 (1.4)	4199 (1.5)	1750 (1.5)
Not recorded	273666 (69.6)	19280 (67.9)	254386 (69.7)	191451 (69.6)	82215 (69.7)
**Smoking Status, n (%)**					
Non-smoker	207007 (52.6)	12715 (44.8)	194292 (53.3)	144885 (52.6)	62122 (52.7)
Ex-smoker	66174 (16.8)	5573 (19.6)	60601 (16.6)	46360 (16.8)	19814 (16.8)
Smoker	77766 (19.8)	6838 (24.1)	70928 (19.4)	54534 (19.8)	23232 (19.7)
Unknown	42282 (10.8)	3276 (11.5)	39006 (10.7)	29480 (10.7)	12802 (10.9)
**Any Alcohol Consumption, n (%)**					
Non-Drinker	367040 (93.3)	26400 (93)	340640 (93.4)	256956 (93.4)	110084 (93.3)
Drinker	16076 (4.1)	1102 (3.9)	14974 (4.1)	11165 (4.1)	4911 (4.2)
Not recorded	10113 (2.6)	900 (3.2)	9213 (2.5)	7138 (2.6)	2975 (2.5)
**Medications, n (%)**					
Statins	28314 (7.2)	3795 (13.4)	24519 (6.7)	19795 (7.2)	8519 (7.2)
AntiCoagulants	6688 (1.7)	1314 (4.6)	5374 (1.5)	4709 (1.7)	1979 (1.7)
AntiDepressants	12275 (3.1)	1622 (5.7)	10653 (2.9)	8548 (3.1)	3727 (3.2)
AntiPsychotics	6582 (1.7)	1324 (4.7)	5258 (1.4)	4629 (1.7)	1953 (1.7)
AntiInflammatories	105431 (26.8)	9315 (32.8)	96116 (26.3)	73885 (26.8)	31546 (26.7)
Steroids	51120 (13)	4958 (17.5)	46162 (12.7)	35875 (13)	15245 (12.9)
**Num. Diagnosis Families, n (%)**					
0	264048 (67.1)	14486 (51)	249562 (68.4)	184564 (67.1)	79484 (67.4)
1	71943 (18.3)	5862 (20.6)	66081 (18.1)	50609 (18.4)	21334 (18.1)
2	30888 (7.9)	3307 (11.6)	27581 (7.6)	21649 (7.9)	9239 (7.8)
3	13947 (3.5)	1961 (6.9)	11986 (3.3)	9793 (3.6)	4154 (3.5)
4	6578 (1.7)	1195 (4.2)	5383 (1.5)	4571 (1.7)	2007 (1.7)
5	3129 (0.8)	729 (2.6)	2400 (0.7)	2164 (0.8)	965 (0.8)
6	1528 (0.4)	460 (1.6)	1068 (0.3)	1079 (0.4)	449 (0.4)
7	680 (0.2)	211 (0.7)	469 (0.1)	477 (0.2)	203 (0.2)
8+	488 (0.1)	191 (0.7)	297 (0.1)	353 (0.1)	135 (0.1)
**Diagnosis Groups, n (%)**					
Respiratory	32719 (8.3)	3584 (12.6)	29135 (8)	22954 (8.3)	9765 (8.3)
Atrial Fibrillation	2975 (0.8)	813 (2.9)	2162 (0.6)	2079 (0.8)	896 (0.8)
Cardiovascular	9185 (2.3)	2206 (7.8)	6979 (1.9)	6423 (2.3)	2762 (2.3)
Osteoarthritis	16170 (4.1)	2263 (8)	13907 (3.8)	11334 (4.1)	4836 (4.1)
Osteoporosis	5011 (1.3)	910 (3.2)	4101 (1.1)	3497 (1.3)	1514 (1.3)
Rheumatoid Arthritis	1716 (0.4)	268 (0.9)	1448 (0.4)	1186 (0.4)	530 (0.4)
Mental Health	45627 (11.6)	5278 (18.6)	40349 (11.1)	32182 (11.7)	13445 (11.4)
Cancer	10067 (2.6)	1544 (5.4)	8523 (2.3)	7038 (2.6)	3029 (2.6)
Digestive	12109 (3.1)	1912 (6.7)	10197 (2.8)	8465 (3.1)	3644 (3.1)
Hypertension	35393 (9)	4568 (16.1)	30825 (8.4)	24841 (9)	10552 (8.9)
Bloodfats	25333 (6.4)	2617 (9.2)	22716 (6.2)	17745 (6.4)	7588 (6.4)
Chronic Kidney Disease	2344 (0.6)	562 (2)	1782 (0.5)	1642 (0.6)	702 (0.6)
Diabetes (type 1)	1336 (0.3)	284 (1)	1052 (0.3)	926 (0.3)	410 (0.3)
Diabetes (type 2)	14476 (3.7)	2403 (8.5)	12073 (3.3)	10092 (3.7)	4384 (3.7)
Venous Thromboembolism	2151 (0.5)	467 (1.6)	1684 (0.5)	1468 (0.5)	683 (0.6)
Other Conditions	2991 (0.8)	520 (1.8)	2471 (0.7)	2107 (0.8)	884 (0.7)

### Outcomes

Table 3 profiles hospitalisations for the Primary Cohort. The rate of emergency or potentially preventable hospitalisations within one year is 7.2%.

**Table 3:** Profiling Hospitalisations of Interest.

	Primary Cohort	
	Count	%
Total Hospitalisations (any duration)	69,183	17.6%
Emergency Hospitalisations within 365 days	26,847	
Potentially Preventable Hospitalisations (PPH) within 365 days	4,514	
“Emergency or PPH” Hospitalisations within 365 days	28,402	7.2%

Note: Nearly 75% of PPH Hospitalisations were also Emergency hospitalisations.

### Model Performance

Table 4 presents a comparison of AUC results using 10-fold cross-validation for the best performing model (the “final” model), a simpler canonical model (age and number of diagnosis families) and a variant on the final model capturing the size of alcohol consumption using the 6-category variable for alcohol per day. (Additional results for the Chronic-only subset are provided in the Supplementary material). Two modelling approaches, Logistic Regression and Generalised boosting are presented as no other method had higher AUC values. Results from 10-fold AUC for these other models were never better than for the final model. The bootstrap estimate of optimism of the final logistic regression model is 0.0017.

**Table 4:** Selected AUC validation results for Logistic Regression and Generalised Boosting.

	Primary Cohort	
	Logistic Regression (95% CI)	Generalised Boosting (95% CI)
Age & Num. Diagnosis Families (both with squared and cubic terms)	0.619 (0.619,0.620)	0.621 (0.621, 0.622)
Final Model, but 6-category alcohol per day instead of “any alcohol”	0.663 (0.663,0.663)	0.666 (0.666,0.666)
Final Model	0.663 (0.663,0.663)	0.666 (0.666,0.666)

As part of the model development process, deviance residuals for the logistic regression models were calculated. For seven variables (age, gender, ethnicity, 6-category BMI, smoking status, any alcohol consumption and SEIFA IRSAD), ANOVA was used to check whether there was a statistically significant relationship between the predictor and the deviance residuals, the presence of which would suggest the model was missing a key relationship. The checks were performed for the residuals of the entire training dataset and for just the Chronic-only subset of the training dataset. A highly significant relationship for gender was found in the Chronic-only subset (P = 0.002) but not in the entire training dataset (P = 0.168). Interaction terms between diagnosis group and gender were investigated by creating an interim model with all diagnosis group-gender interaction terms. Interaction terms in the model were retained for the final model if the P-value for the interaction term in the model output was below a generous threshold of 0.5. As a result, 9 interaction terms were included in the final model. Repeating the ANOVA checks for the final model, inclusion of these 9 terms largely removed the effect for gender (training dataset P = 0.043, Chronic-only P = 0.052). P-values for other variables were always larger than 0.03 for both datasets before and after adding the interaction terms.

**Figure 3:**
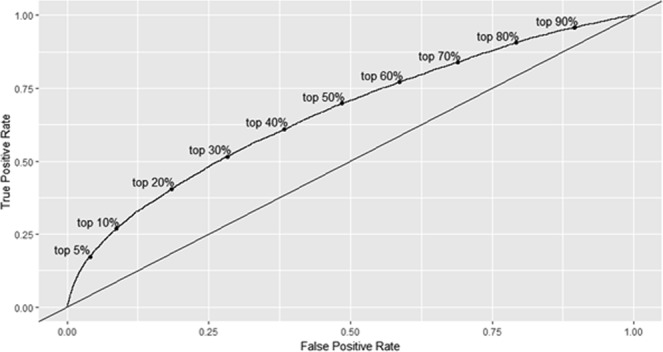
Receiver-operating characteristic curve for final logistic model (AUC = 0.66). Note: Risk groups are presented by deciles.

**Figure 4:**
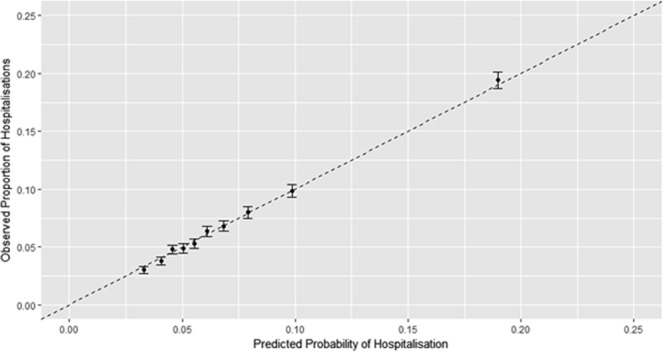
Calibration curve for risk groups by deciles of predicted risk: final logistic model.

Figure 3 shows the receiver operating characteristic (ROC) curve for the final model. Labelled points indicate the corresponding location of risk for indicated quantiles. The diagonal reference line is the line of no discrimination and corresponds to the performance of random guesses. Figure 4 shows a calibration curve for risk groups defined by deciles of predicted risk. For each group, the horizontal coordinate is the mean of predicted risk of hospitalisation and the vertical coordinate is the mean (with 95% confidence interval) for the observed proportion of hospitalisations. The dashed diagonal line indicates ideal performance where observed and predicted risk are equal.

Using a 70%/30% train/test data split, we checked the amount of bias in our unknown/not recorded categories by stratifying the test dataset into known and unknown parts for variables of interest (BMI, ethnicity, smoking, alcohol consumption, IRSAD advantage/disadvantage). Predictive performance on the 30% test dataset (AUC 0.665) is similar to that from 10-fold cross validation (AUC 0.663), so the results should be indicative. Calibration for the known parts are universally excellent. For the unknown parts, if there is bias it is small. Comparing mean predictions to observed proportions of hospitalisations, all results are within confidence intervals (BMI mean prediction 0.184, observed 0.190 (95% CI: 0.181–0.199; ethnicity mean prediction 0.212, observed 0.218 (95% CI: 0.202–0.234), smoking mean prediction 0.214, observed 0.233 (95% CI: 0.211–0.255); IRSAD unknown or AnyAlcohol not recorded mean prediction 0.232, observed 0.264 (95% CI: 0.228–0.300). For all of these results, the difference in mean with the neighbouring risk group decile greatly exceeds the difference between known/recorded and unknown/not recorded within the decile.

### Model Specification and Computation

Table 5 shows the complete model specification for the final logistic model. The model coefficient for each predictor is shown in column 2. For categorical predictors, coefficients are for 0/1 binary indicator variables (dummy coding). With dummy coding, each category (except the reference category) gets its own binary variable, where 1 indicates the categorical predictor takes that value. Columns 3–4 shows the standard error and P-value for each coefficient. Column 5 shows the odds ratio with 95% confidence interval for each category compared to its reference level. Odds ratios are not provided for continuous predictors as their interpretation is made more complicated by quadratic and higher order terms.

**Table 5:** Full Specification: Final Logistic Model.

i	Variable (V_i_)	Coefficient (C_i_)	Std. Error	Odds Ratio (95% CI)
	Intercept	−2.7551480		
1	Age	−0.0379528	0.00663	
2	Age^2^	6.15945E-04	0.00014	
3	Age^3^	−1.04739E-06	8.77732E-07	
	Gender			
4	Female	0.2216384	0.01588	1.248 (1.210,1.288)
	Ethnicity			
5	Indigenous Australian	0.4033629	0.07675	1.497 (1.288,1.740)
6	Unknown	−0.0662497	0.01660	0.936 (0.906,0.967)
	BMI			
7	[25, 30)	0.0329420	0.02884	1.033 (0.977,1.094)
8	[30, 35)	0.1939825	0.03225	1.214 (1.140,1.293)
9	[35, 40)	0.2712273	0.04200	1.312 (1.208,1.424)
10	40+	0.4643618	0.04627	1.591 (1.453,1.742)
11	Not recorded	0.1395070	0.02198	1.150 (1.101,1.200)
	Smoking Status			
12	Ex smoker	0.1969316	0.01761	1.218 (1.176,1.260)
13	Smoker	0.4057213	0.01636	1.500 (1.453,1.549)
14	Unknown	0.1993470	0.02421	1.221 (1.164,1.280)
	AnyAlcohol			
15	Drinker	−0.2853232	0.03423	0.752 (0.703,0.804)
16	Not recorded	−0.2301394	0.04245	0.794 (0.731,0.863)
	SEIFA IRSAD			
17	1	−0.0123849	0.02505	0.988 (0.940,1.037)
18	2	−0.0742180	0.05930	0.928 (0.827,1.043)
19	3	0.0044308	0.03370	1.004 (0.940,1.073)
20	4	−0.1857272	0.02472	0.831 (0.791,0.872)
21	6	−0.2340966	0.02585	0.791 (0.752,0.832)
22	7	−0.2468854	0.02507	0.781 (0.744,0.821)
26	8	−0.2990624	0.02832	0.742 (0.701,0.784)
24	9	−0.1834029	0.02176	0.832 (0.798,0.869)
25	10	−0.4622169	0.02792	0.630 (0.596,0.665)
26	Unknown	−0.0301620	0.13850	0.970 (0.740,1.273)
	Medications			
27	Statins	−0.0152881	0.02606	0.985 (0.936,1.036)
28	AntiCoagulants	0.2886091	0.04044	1.335 (1.233,1.445)
29	AntiDepressants	0.2025163	0.03020	1.224 (1.154,1.299)
30	AntiPsychotics	0.3923084	0.03527	1.480 (1.382,1.586)
31	AntiInflammatories	0.1301034	0.01442	1.139 (1.107,1.172)
32	Steroids	0.1488350	0.01800	1.160 (1.120,1.202)
	Number of Diagnosis Families			
33	Num. Diagnosis Families	0.3369661	0.03298	
34	(Num. Diagnosis Families)^2^	−0.0397663	0.00653	
35	(Num. Diagnosis Families)^3^	0.0019304	0.00057	
	Diagnosis Groups			
36	Respiratory	−0.0715037	0.04264	0.931 (0.856,1.012)
37	Atrial Fibrillation	0.2234789	0.05779	1.250 (1.117,1.400)
38	Cardiovascular	0.4764327	0.04877	1.610 (1.464,1.772)
39	Osteoarthritis	−0.2060183	0.03764	0.814 (0.756,0.876)
40	Osteoporosis	0.0595034	0.08957	1.061 (0.890,1.265)
41	Rheumatoid Arthritis	0.1149149	0.07502	1.122 (0.968,1.299)
42	Mental Health	0.0686955	0.04483	1.071 (0.981,1.169)
43	Cancer	0.0600825	0.04073	1.062 (0.980,1.150)
44	Digestive Diseases	0.1796635	0.03992	1.197 (1.107,1.294)
45	Hypertension	−0.1591489	0.04100	0.853 (0.787,0.924)
46	Bloodfats	−0.3726723	0.03771	0.689 (0.640,0.742)
47	Chronic Kidney Disease	0.0268266	0.08500	1.027 (0.870,1.213)
48	Diabetes (type 1)	0.5844975	0.10630	1.794 (1.457,2.210)
49	Diabetes type 2)	0.1332004	0.04722	1.142 (1.041,1.253)
50	Venous Thromboembolism	0.3623621	0.06477	1.437 (1.265,1.631)
51	Other Conditions	0.5157983	0.08211	1.675 (1.426,1.967)
	Pathology Test Results			
	Haemoglobin (g/L)			
52	High (<100)	0.4416708	0.06741	1.555 (1.363,1.775)
53	Med (M:100–130, F: 100–120)	0.1546069	0.02639	1.167 (1.108,1.229)
54	No test history	0.0450222	0.17061	1.046 (0.749,1.461)
	Platelets (per L)			
55	High (>480 × 1e9)	0.1398411	0.08950	1.150 (0.965,1.371)
56	No test history	−0.0147097	0.17048	0.985 (0.706,1.376)
	Alanine aminotransferase level (u/L)			
57	High (M: >120, F: >90)	−0.0619518	0.09056	0.940 (0.787,1.122)
58	Med (M: 80–120, F: 60–90)	0.0550267	0.06484	1.057 (0.930,1.200)
29	No test history	−0.2940664	0.24315	0.745 (0.463,1.200)
	Gamma-glutamyl transferase (u/L)			
60	High (M: >150, F: >105)	0.2209109	0.04884	1.247 (1.133,1.372)
61	Med (M: 100–150, F 70–105)	0.1261477	0.04895	1.134 (1.031,1.249)
62	No test history	0.1979201	0.21908	1.219 (0.793,1.873)
	Haemoglobin A1c level (mmol/mol)			
63	High (〉〉69.4)	0.1746667	0.04153	1.191 (1.098,1.292)
64	Med (58.5–69.4)	0.1720256	0.05061	1.188 (1.076,1.312)
65	No test history	−0.0461721	0.01882	0.955 (0.920,0.991)
	Total cholesterol (mmol/L)			
66	High (>7.5)	0.0453054	0.07726	1.046 (0.899,1.217)
67	Med (6.5–7.5)	−0.0363534	0.04640	0.964 (0.880,1.056)
68	No test history	0.1749709	0.15300	1.191 (0.883,1.608)
	Albumin/creatinine ratio (mg/mmol)			
69	High (>30)	0.3042750	0.08592	1.356 (1.146,1.604)
70	Med (3–30)	0.1151562	0.05232	1.122 (1.013,1.243)
71	No test history	0.1059512	0.02951	1.112 (1.049,1.178)
	LDL cholesterol (mmol/L)			
72	High (>4)	−0.0585925	0.03821	0.943 (0.875,1.016)
73	Med (3–4)	−0.0539600	0.02062	0.947 (0.910,0.987)
74	No test history	0.1556545	0.05693	1.168 (1.045,1.306)
	Estimated glomerular filtration rate (ml/min)			
75	High (<30)	0.0891486	0.07968	1.093 (0.935,1.278)
76	Med (30–45)	0.0950474	0.05205	1.100 (0.993,1.218)
77	No test history	0.0459790	0.02027	1.047 (1.006,1.089)
	Blood pressure			
78	High (systolic >160 and diastolic >100)	0.2946162	0.10197	1.343 (1.099,1.640)
79	Med (systolic: 140–160 AND diastolic: 90–100)	0.1839175	0.03706	1.202 (1.118,1.292)
80	No test history	0.0112105	0.01543	1.011 (0.981,1.042)
	Bilirubin (umol/L)			
81	Med_or_High (>40)	0.1803894	0.15242	1.198 (0.888,1.615)
82	No test history	0.0784321	0.21276	1.082 (0.713,1.641)
	Creatinine (umol/L)			
83	Med_or_High (M: >350, F: >300)	1.1349460	0.15125	3.111 (2.313,4.185)
84	No test history	−0.1704331	0.02109	0.843 (0.809,0.879)
	Triglycerides (mmol/L)			
85	Med_or_High (>4)	0.1234874	0.05878	1.131 (1.008,1.270)
86	No test history	−0.0963018	0.16070	0.908 (0.663,1.244)
	Gender-Diagnosis Group Interactions Terms			
87	Gender × Respiratory	0.0570566	0.04067	1.059 (0.978,1.147)
88	Gender × Cardiovascular	−0.2108839	0.05868	0.810 (0.722,0.909)
89	Gender × Osteoporosis	−0.2475655	0.09695	0.781 (0.646,0.944)
90	Gender × Mental health	−0.0643675	0.03514	0.938 (0.875,1.005)
91	Gender × Hypertension	−0.0890935	0.04019	0.915 (0.845,0.990)
92	Gender × Chronic Kidney disease	−0.1122160	0.10963	0.894 (0.721,1.108)
93	Gender × Diabetes (type 1)	0.2492323	0.14198	1.283 (0.971,1.695)
94	Gender × Diabetes (type 2)	−0.0002784	0.05227	1.000 (0.902,1.108)
95	Gender × Other Conditions	−0.1721620	0.10383	0.842 (0.687,1.032)

Note: Coefficients for Age^2^ and Age^3^ are shown using scientific notation to provide additional precision.

As with other logistic regression models, predictions are calculated via the linear predictor. The linear predictor, *y*, is defined using the equation

$$y={\rm{Intercept}}+\,\sum _{i=1}^{N}({C}_{i}\times {V}_{i})$$

where for each *i*, *C*_*i*_ is the model coefficient and *V*_*i*_ is the corresponding variable value (e.g., 0 or 1 for binary indicator and dummy coding variables). The value of the intercept is also shown in the table. Note that for this model, $N=95$. The predicted probability, *p*, is calculated from the linear predictor according to the equation

$$p=\exp (y)/(1+\exp (y))$$

which necessarily satisfies $0\le p\le 1$.

Five test cases showing values of the predictor variables, the linear predictor and the predicted probability are presented in the Supplementary material to support computational reproducibility.

## Discussion

This study presents a prediction model designed for Australian primary care practices to identify patients with chronic conditions in their patient population that are at high risk of hospitalisation over the next 12 months. Despite not having access to a rich linked data repository and high impact predictors such as previous hospitalisation history, the prediction model performs at similar levels to other state-of-the-art prediction models and demonstrates the efficacy of using this approach in similar settings.

Our model shows good performance by the area under the Receiver Operating Characteristic curve (AUC~0.66) and is well calibrated. As might be expected, obesity and smoking increase the risk of hospitalisation, while higher economic advantage lowers the risk of hospitalisation. Interestingly, alcohol consumption is also associated with decreased risk of hospitalisation. However, in our cohort many more drinkers have trivial to moderate consumption, so we would expect the category to be dominated by these kinds of drinkers. QAdmissions^8^ reported decreased risk of hospitalisations for this group, compared with increased risk for heavy drinkers, so our model appears consistent with other published results. We further refined our focus to the subset of patients with at least one diagnosed chronic condition, for which targeted healthcare is more important. On this subset, our model showed even better performance. Smoothed plots of predicted vs observed probabilities suggest that our model is doing well to capture increasing risk by age and number of diseases, two variables known to be important for this kind of risk stratification.

In current literature, variables related to prior health care utilisation have been shown to have a strong influence on the performance of such models. A review by Wallace *et al*.^9^ explored 18 risk prediction models that used routinely collected data for prediction. Seven of the 8 models in this group that were comparable to this study employed prior emergency admission in the final model, while the eighth created customised models that used a combination of data sources that included hospitalisation and community data. For example, the QAdmissions^8^ algorithm predicts emergency admission within 2 years (AUC 0.77–0.78) but, like most other algorithms, employs prior emergency hospitalisation information (sourced from both GP data and linked hospital episode data) as the most significant predictor.

Heterogeneity in the definitions of study populations and outcome measures limits the ability to directly compare our model’s performance with other work. Models such as the adapted PEONY^21^ and HARP^22^, and the models presented by Johnson *et al*.^23^ focus on older patients only and are therefore not directly comparable. In addition, while Johnson *et al*. present models based on “GP-like-data”, data for their study is drawn from a self-reported, English-only, voluntary survey and simulates conditions where data is accessed through primary care practice management systems. It is therefore likely to be more complete than real-world primary care data. Also, they use survey variables like ‘health condition count’ and ‘self-rated health’ to approximate the patient health history information that is commonly captured in GP patient management software. Our experience of data extracted directly from GP patient management systems suggests that this is far from representative of the true picture. Haas *et al*.^24^ evaluated the performance of six algorithms in predicting outcome measures including inpatient hospitalisation (AUC 0.67–073) and Emergency Department visits (AUC 0.58 to 0.67) within 12 months. While somewhat similar, both of these outcome measures are different enough from the one employed for this study to make direct comparison difficult. On the other hand, efforts such as the Gold Coast Integrated Care in Australia^25^ employ a purposely designed Risk of Hospitalisation scoring mechanism for which no validation or performance information is published, making comparison impossible. In general, when comparing similar outcome measures, our predictive risk model performed at a level that was similar to, if not better than, other models that did not include previous hospitalisation as a predictor variable.

As with other studies of this type, there are limitations to this work. We suspect that model performance was constrained by primary care data quality issues, especially data correctness, inconsistencies, and completeness. Significant effort was devoted to data preparation to ensure quality issues (e.g., data entry issues, unit inconsistencies) were appropriately handled, derived predictors were calculated in a consistent manner, and steps were taken to reduce missingness where possible (e.g., calculating BMI where height and weight could be found in the data). Nevertheless, missing data was a feature in several key variables (BMI, ethnicity, alcohol consumption, smoking history, index of advantage/disadvantage). The expected use for our model is in GP clinics using computerised systems similar to those from which the sample data was obtained. Thus, we anticipate similar patterns of missingness in the production environment.

Statistical approaches to missing data include complete case analysis and multiple imputation. We avoided complete case analysis due to concerns about introducing bias. Multiple imputation is a common approach but requires an assumption that the data is “missing at random” and development of additional models to impute missing data, possibly using additional covariates. We deliberately used a different approach, creating an unknown or “not recorded” category for each of these categorical predictors. We believe that in many cases the missingness reflects the nature of the missing value and possibly the reason for the GP visit. In short, we believe the data is missing not at random and has predictive power of its own. An advantage of our approach is that it easily extends to a production environment in which it is necessary to make predictions with data that similarly exhibits missingness. Unfortunately, the approach can also introduce bias. In our estimates that bias is small, but it means care should be exercised concerning the interpretation of model coefficients and standard errors for those predictors in particular. What remains clear is that complete data is preferable to missing data for these kinds of models, and we hope that data completeness in these key predictors increases.

Due to limitations in recording processes and available data fields, clinically relevant semantics of medication and diagnosis variables were not considered. For example, mode of delivery and dosage were not incorporated into medication predictors, diagnosis variables did not distinguish between treated and untreated conditions/diseases, and medications were not ascribed to particular conditions/diseases. We leave the study of such predictors for future work. Also, our choice to include potentially preventable hospitalisations in the response variable was guided by studies^11,12^ recommending the targeting of potentially preventable hospitalisations through integrated care and other reform measures. We recognise that not all such hospitalisations are actually preventable in the short-term through management in primary care^10^. In practice, the addition of clinical assessment of patients with high probability of hospitalisation would allow further tailoring of primary care appropriate to patient needs.

Our prediction model provides a validated tool for risk stratification in GP patient populations and is deployed within a software suite used in the Health Care Homes trial. It is implemented in a number of general practice clinics (up to 200) within a web application which sources data from five different general practice information systems and uses the model to identify patients at high risk of hospitalisation over the following 12 months. Combined with other elements of the Health Care Homes trial, which include a clinical assessment to assign identified patients to tiers and a patient-centric integrated care program, it is hoped that this model will help deliver significant improvements to patient outcomes while reducing the burden of chronic conditions on the Australian health system.

## Supplementary information

Supplementary Information

## Data Availability

The datasets analysed in the current study are not publicly available. Due to reasonable privacy and security concerns and requirements imposed by the ethics approval process, they are not redistributable to researchers other than those engaged in the ethics committee approved research protocol.
