# Supplementary material for: A risk stratification tool for hospitalisation in Australia using primary care data
Source: Sci Rep. 2019 Mar 21;9:5011. doi: 10.1038/s41598-019-41383-y (PMC6428894; doi:10.1038/s41598-019-41383-y)
Supplement: Supplementary file 1 — Supplementary Information [file 41598_2019_41383_MOESM1_ESM.docx]

**Supplementary Information**

**A risk stratification tool for hospitalisation in Australia using primary care data**

Sankalp Khanna, David A. Rolls, Justin Boyle, Yang Xie, Rajiv Jayasena, Marienne Hibbert, Michael Georgeff

Supplementary Tables

Supplementary Table S1 - Medication groups and corresponding regular search (regex) expressions

| **Medication Group** | **Regex** |
| --- | --- |
| 'STATINS' | .*(Amlodipine besylate\|Atorvastatin\|Atorvastatin([^A-Z0-9]){0,3}Amlodipine\|Avena Sativa\|Cerivastatin\|Cholestyramine\|Clofibrate\|Colestipol\|Colestipol Hydrochloride\|Crestor\|Ezetimibe\|Ezetimibe([^A‑Z0‑9]){0,3}Simvastatin\|Fenofibrate\|Fluvastatin\|Gemfibrozil\|Lescol\|Lipitor\|Nicotinic Acid\|Policosanol\|Pravachol\|Pravastatin\|Pravastatin Sodium\|Probucol\|Rosuvastatin\|Simvastatin\|Zocor).*' |
| 'ANTICOAGULANTS' | '.*(apixaban\|Arixtra\|Clexane\|Coumadin\|dabigatran\|Daktarin\|dalteparin\|Eliquis\|enoxaparin\|fondaparinux\|Fragmin\|heparin\|Marevan\|Pradaxa\|rivaroxaban\|warfarin\|Xarelto).*' |
| 'ANTIDEPRESSANTS' | '.*(agomelatine\|amitriptyline\|citalopram\|clomipramine\|desvenlafaxine\|dothiepin\|doxepin\|duloxetine\|escitalopram\|fluovoxamine\|fluoxetine\|imiprimine\|mianserin\|mirtazapine\|moclobemide\|nortriptyline\|paroxetine\|phenelzine\|reboxetine\|sertraline\|tranylcypromine\|trimipramine\|venlafaxine).*' |
| 'ANTIPSYCHOTICS' | '.*(Abilify\|Amipride\|amisulpride\|Anatensol\|Anxiostat\|aripiprazole\|asenapine\|Chlorpromazine\|Clopine\|Clopixol\|CloSyn\|Clozapine\|Clozaril\|DBL\|Deprel\|Droleptan\|Droperidol\|Fluanxol\|flupenthixol\|fluphenazine\|Haldol\|haloperidol\|Invega\|Largactil\|Lithicarb\|Lithium\|lurisidone\|Modecate\|Neulactil\|olanzapine\|Orion\|paliperidone\|pericyazine\|quetiapine\|Quilonum\|Rispa\|Risperdal\|risperidone\|Rixadone\|Serenace\|Seroquel\|Solian\|Stelazine\|trifluoperazine\|Zeldox\|ziprasidone\|zuclopenthixol\|Zyprexa).*' |
| 'ANTI_INFLAMMATORY' | '.*(Aclin\|ACT3\|Actiprofen\|Actron\|Acular\|Advil\|Aleve\|Anaprox\|Arthrexin\|Arthrotec\|Brufen\|Bugesic\|Celebrex\|celecoxib\|Clinoril\|Clonac\|Codeine\|Codeine Phosphate\|Crysanal\|Diclac\|Diclofenac\|Diclofenac‑BC\|Diclohexal\|diflunisal\|Dinac\|Dolobid\|Eazydayz\|Feldene\|Fenac\|Hexal\|Ibuprofen\|Ibuprofen Lysine\|Imflac\|Indocid\|Indomethacin\|Inza\|Ketoprofen\|Ketorolac\|Ketorolac Trometamol\|Mefenamic acid\|Mefic\|meloxicam\|Misoprostol\|Mobic Vivlodex\|Mobilis\|nabumetone\|Naprogesic\|Naprosyn\|Naproxen\|Nurofen\|Nurolasts\|Orudis\|Oruvail\|Panafen\|Pirohexal‑D\|Piroxicam\|Ponstan\|Proven\|Proxen\|Pseudoephedrine Hydrochloride\|Rafen\|Relafen\|Rosig\|Solaraze\|Sulindac\|Surgam\|Tiaprofenic Acid\|Toradol\|Tri-Profen\|Voltaren\|Voltfast\|Trometamol).*' |
| 'STEROIDS' | '.*(Beclometasone dipropionate\|Budesonide\|Ciclesonide\|Fluticasone furoate\|Fluticasone propionate\|Hydrocortisone\|Methylprednisolone sodium succinate\|prednisolon\|prednisone).*' |

Supplementary Table S2 – Defining Diagnosis Families and Diagnosis Groups

| **Diagnosis Group** | **Diagnosis Family** | **Diagnosed Conditions/Diseases Included** |
| --- | --- | --- |
| **diagnosisgrp.respiratory.flag** | diagnosis.asthma.flag | asthma |
|  | diagnosis.copd.flag | chronic obstructive pulmonary disease, chronic obstructive airways disease, chronic bronchitis, emphysema, chronic asthma, bronchiectasis |
| **diagnosisgrp.atrial_fibr.flag** | diagnosis.atrial_fibr.flag | Atrial fibrillation |
| **diagnosisgrp.cardiovascular.flag** | diagnosis.coronary_heart.flag | Atherosclerotic heart disease, Coronary artery spasm/surgery/stent/blockage, angiography/angioplasty, coronary endarterectomy/occlusion/insufficiency, acute coronary syndrome, Coronary bypass, ischaemic heart disease, angina, heart attack, Atherosclerosis, Myocardial damage, myocardial insufficiency, Percutaneous transluminal angioplasty, Subendocardial infarction |
|  | diagnosis.stroke.flag | Cerebrovascular disease, Cerebellar infarction/embolism/haemorrhage |
|  | diagnosis.tia.flag | Transient ischemic attack |
|  | diagnosis.cong_heart_failure.flag | Congestive heart failure, left/right ventricular failure, Cardiomyopathy, Pulmonary oedema |
|  | diagnosis.rheumatic_heart.flag | rheumatic heart disease |
| **diagnosisgrp.osteoarthritis.flag** | diagnosis.osteoarthritis.flag | Osteoarthritis |
| **diagnosisgrp.osteoporosis.flag** | diagnosis.osteoporosis.flag | Osteoporosis |
| **diagnosisgrp.rheumatoid.flag** | diagnosis.rheumatoid.flag | Rheumatoid arthritis |
| **diagnosisgrp.mental_health.flag** | diagnosis.depression.flag | Depression |
|  | diagnosis.anxiety.flag | Anxiety |
|  | diagnosis.bipolar.flag | Bipolar, Manic Depression |
|  | diagnosis.schizophrenia.flag | Schizophrenia |
|  | diagnosis.dementia.flag | Dementia, Alzheimer's disease |
|  | diagnosis.learning_diff.flag | Learning difficulties |
| **diagnosisgrp.cancer.flag** | diagnosis.cancer.flag | Cancer , Carcinoma, Melanoma, Lymphoma |
| **diagnosisgrp.digestive.flag** | diagnosis.crohns.flag | Crohns disease |
|  | diagnosis.ulcer_colitis.flag | Ulcerative colitis, inflammatory bowel disease |
|  | diagnosis.coeliac.flag | Coeliac disease |
|  | diagnosis.steatorrhea.flag | Steatorrhea |
|  | diagnosis.malabsorp_syndr.flag | Intestinal malabsorption, Malabsorption syndrome, fructose malabsorption |
|  | diagnosis.chronic_liver.flag | Liver cirrhosis/dysfunction/failure/fibrosis, hepatic disease |
|  | diagnosis.pancreatitis.flag | Pancreatitis, Pancreatic insufficiency |
| **diagnosisgrp.hypertension.flag** | diagnosis.hypertension.flag | Hypertension |
| **diagnosisgrp.bloodfats.flag** | diagnosis.hyperlipidaemia.flag | Hyperlipidaemia |
|  | diagnosis.hypercholesterolaemia.flag | Hypercholesterolaemia |
|  | diagnosis.hypertriglyceridaemia.flag | Hypertriglyceridaemia |
| **diagnosisgrp.chronic_kidney.flag** | diagnosis.chronic_kidney.flag | chronic kidney disease, chronic renal failure/insufficiency, chronic glomerulonephritis, end stage renal disease, renal impairment, dialysis, Macroalbuminuria, Macroproteinuria, Proteinuria |
| **diagnosisgrp.diabetes_type_1.flag** | diagnosis.diabetes_type_1.flag | Diabetes mellitus type 1 |
| **diagnosisgrp.diabetes_type_2.flag** | diagnosis.diabetes_type_2.flag | Diabetes mellitus type 2 |
| **diagnosisgrp.venous_thrombo.flag** | diagnosis.venous_thrombo.flag | Venous thromboembolism, Pulmonary embolism, DVT |
| **diagnosisgrp.other.flag** | diagnosis.falls.flag | Fall |
|  | diagnosis.epilepsy.flag | Epilepsy |

All diagnosis family variables are 0/1 with 1 indicating a diagnosis, and 0 otherwise. All diagnosis group variables are 1 if at least one corresponding diagnosis family variable is 1, and 0 otherwise.

Supplementary Table S3 - Morbidity risk groupings for pathology/physiological observations

| **Pathology / Physiological Observations** | **Moderate Morbidity** | **Higher Morbidity** | **LOINC** |
| --- | --- | --- | --- |
| GGT, GGT/ Gamma GT/  S Gamma-GT | If M > 2*50 (u/L)  If F > 2*35 (u/L) | If M > 3*50 (u/L)  If F > 3*35 (u/L) | 2324-2 |
| Bilirubin | >2*20 (umol/L) | > 3*20 (umol/L) | 14631-6 |
| ALT, ALT/ S ALT/ Alanine Aminotransferase | If M > 2*40 (u/L) ,  If F > 2*30 (u/L) | If M > 3*40 (u/L)  If F > 3*30 (u/L) | 1742-6 |
| Platelets |  | > 480 (x 10^9)^/L | 777-3 |
| HbA1c | > 58.5 mmol/mol | > 69.4 mmol/mol | 4548-4 |
| ACR (Albumin/ creatinine ratio) | If >3 mg/mmol | If > 30 mg/mmol | 14959-1 |
| Total Cholesterol | > 6.5 mmol/L | > 7.5 mmol/L | 14647-2 |
| BP | > 140/90 mmHg | >160/100 mmHg |  |
| creatinine/ s creatinine | If M >= 350 umol/L  if F >= 300 umol/L | If M >= 2*350 umol/L  if F >= 2*300 umol/L | 14682-9 |
| eGFR | 30 ml/min < x <45 ml/min | <30 ml/min | 33914-3 |
| Triglycerides | >4 mmol/L | >8 mmol/L | 14927-8 |
| Haemoglobin | If M < 130 g/L  If F < 120 g/L | <=100g/L | 718-7 |
| LDL | 3 mmol/L < x < 4.0 mmol/L | > 4.0 mmol/L | 22748-8 |

For gender-specific criteria, M (F) indicates male (female).

Supplementary Results for the Chronic-Only Subset

The subgroup of patients with at least one chronic condition (Chronic-only subset) is an important subgroup of primary care patients. Here we present results using the final logistic regression model to predict the risk of hospitalisation for this subset of patients.

Supplementary Table S4 profiles hospitalisations in the Chronic-only subset of patients. The rate of hospitalisation in this group, at 11%, is slightly higher than 7% for the larger Primary cohort. This is consistent with the general idea of people with chronic conditions requiring more acute care.

Supplementary Table S4 - Profile of Hospitalisations in the Chronic-only Subset

|  | **Chronic-Only Subset** | |
| --- | --- | --- |
|  | **Count** | **%** |
| **Total Hospitalisations (any duration)** | 31,449 | 24.40% |
| **“Emergency or PPH” Hospitalisations within 365 days** | 13,884 | 10.80% |

Validation results for area under the ROC curve using 10-fold cross-validation for the Chronic-Only subset are shown in Supplementary Table S5. These results involved fitting the model to all the data in nine “training” folds and using the Chronic-only subset of the tenth “test fold” for validation, repeating for each fold, and combining results across folds. Again, results for the final logistic regression and generalised boosting model are similar. Also, the performance of models that capture volume of alcohol consumed are similar to those that treat alcohol consumption in a yes/no fashion.

Supplementary Table S5 - Selected AUC validation results for Logistic Regression and Generalised Boosting using the Chronic-only subset

|  | **Chronic-only Subset** | |
| --- | --- | --- |
|  | **Logistic Regression**  **(95% CI)** | **Generalised Boosting**  **(95% CI)** |
| Age & Num Diagnosis Families  (both with squared and cubic terms) | 0.614 (0.614,0.615) | 0.618 (0.618, 0.618) |
| Final Model, but 6-category alcohol per day instead of “any alcohol” | 0.675 (0.675, 0.675) | 0.678 (0.678,0.678) |
| Final Model | 0.675 (0.675, 0.675) | 0.678 (0.678,0.678) |

For additional results obtained with a 70%/30% train/test split, the model was fit to the 70% Primary cohort training data and the Chronic-only subset of patients in the 30% “Test” data was used as the test dataset. This Chronic-Only “Test” subset comprised 38,373 patients. Supplementary Table S6 shows various performance measures of the prediction model at various threshold levels. The corresponding receiver-operating characteristic curve is shown in Supplementary Figure S1.

Supplementary Figure S2 shows a calibration curve for risk groups defined by deciles of predicted risk. For each group, the horizontal coordinate is the mean of predicted risk of hospitalisation and the vertical coordinate is the mean (with 95% confidence interval) for the observed proportion of hospitalisations.

Supplementary Table S6 – Model Performance – Chronic Condition Test Cohort

| **Description** | **Top 5%** | **Top 10%** | **Top 20%** | **Top 30%** | **Top 40%** | **Top 50%** | **Top 60%** |
| --- | --- | --- | --- | --- | --- | --- | --- |
| True Positives | 664 | 1133 | 1743 | 2235 | 2637 | 2991 | 3306 |
| False Positives | 1239 | 2764 | 6003 | 9427 | 12831 | 16378 | 19860 |
| True Negatives | 32979 | 31454 | 28215 | 24791 | 21387 | 17840 | 14358 |
| False Negatives | 3491 | 3022 | 2412 | 1920 | 1518 | 1164 | 849 |
| Multi Chronic Condition Patients in True Cohort | 1828 | 3602 | 6423 | 8718 | 10566 | 12182 | 13503 |
| Actual True Cases | 4155 | 4155 | 4155 | 4155 | 4155 | 4155 | 4155 |
| Actual False Cases | 34218 | 34218 | 34218 | 34218 | 34218 | 34218 | 34218 |
| Predicted True Cases | 1903 | 3897 | 7746 | 11662 | 15468 | 19369 | 23166 |
| Predicted False Cases | 36470 | 34476 | 30627 | 26711 | 22905 | 19004 | 15207 |
| Sensitivity | 16.0% | 27.3% | 41.9% | 53.8% | 63.5% | 72.0% | 79.6% |
| Specificity | 96.4% | 91.9% | 82.5% | 72.5% | 62.5% | 52.1% | 42.0% |
| Positive Predictive Value | 34.9% | 29.1% | 22.5% | 19.2% | 17.0% | 15.4% | 14.3% |
| Negative Predictive Value | 90.4% | 91.2% | 92.1% | 92.8% | 93.4% | 93.9% | 94.4% |
| Accuracy | 87.7% | 84.9% | 78.1% | 70.4% | 62.6% | 54.3% | 46.0% |
| Multi Chronic Condition Patients in True Cohort (%) | 96.1% | 92.4% | 82.9% | 74.8% | 68.3% | 62.9% | 58.3% |

Supplementary Figure S3 and Supplementary Figure S4 present the Predicted Probability of Hospitalisation by Age and by Number of Diagnoses (i.e. total number of distinct diagnosis families), respectively, for the Chronic-only subset of the Test dataset. Each blue dot shows the predicted risk of hospitalisation for one patient in the Chronic-only subset of the Test dataset. The black curve shows a smooth curve through the blue points, with a grey 95% confidence envelope. The red line shows a smooth curve through the observed hospitalisations (using 0/1 binary indicator variables) with a grey 95% confidence envelope. Both Age and by Number of Diagnoses are important for stratifying patient risk and these figures illustrate how the model captures the variation in risk, on average, for these two predictors. In particular, predicted probabilities (black curves) track observed probabilities (red curves) quite closely except at extreme values on the right where incidence is sparse.


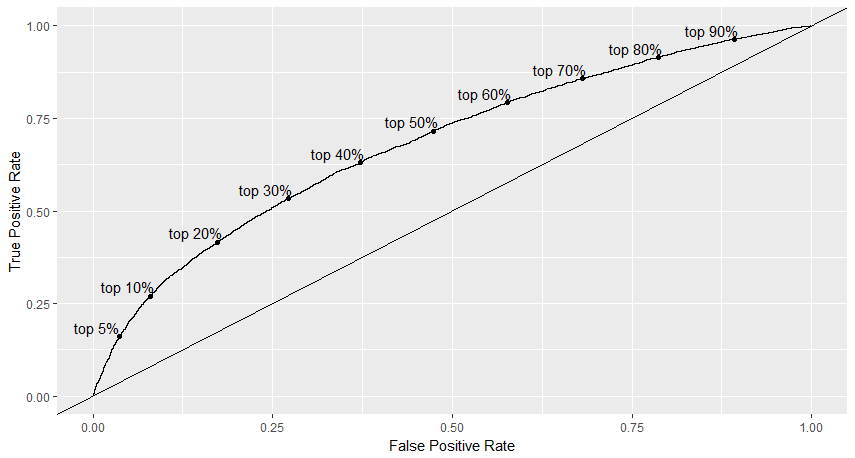


Supplementary Figure S1 – Receiver-operating characteristic (ROC) curve for the final model using the Chronic-only subset


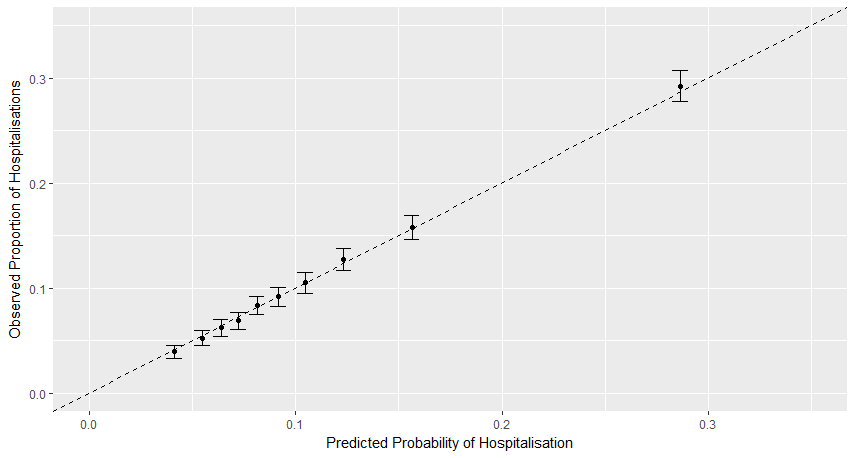


Supplementary Figure S2 - Calibration curve for risk groups by deciles of predicted risk using the Chronic-only subset


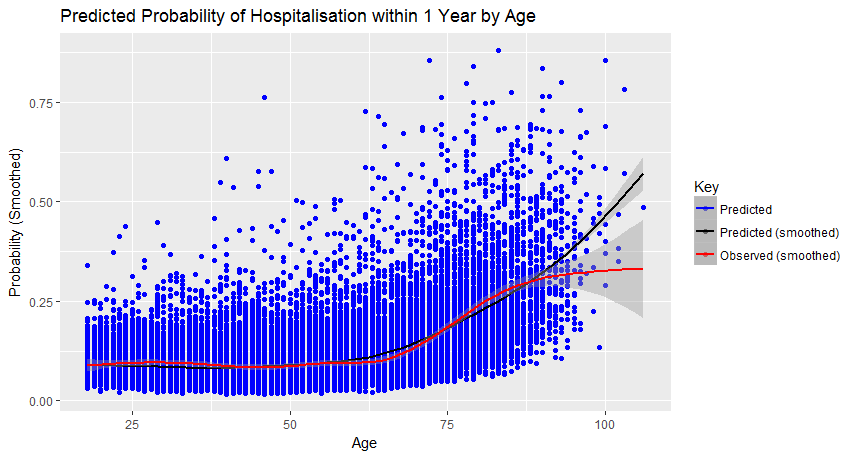


Supplementary Figure S3 – Predicted Probability of Hospitalisation within 1 Year by Age for the Chronic-only subset of the Test dataset


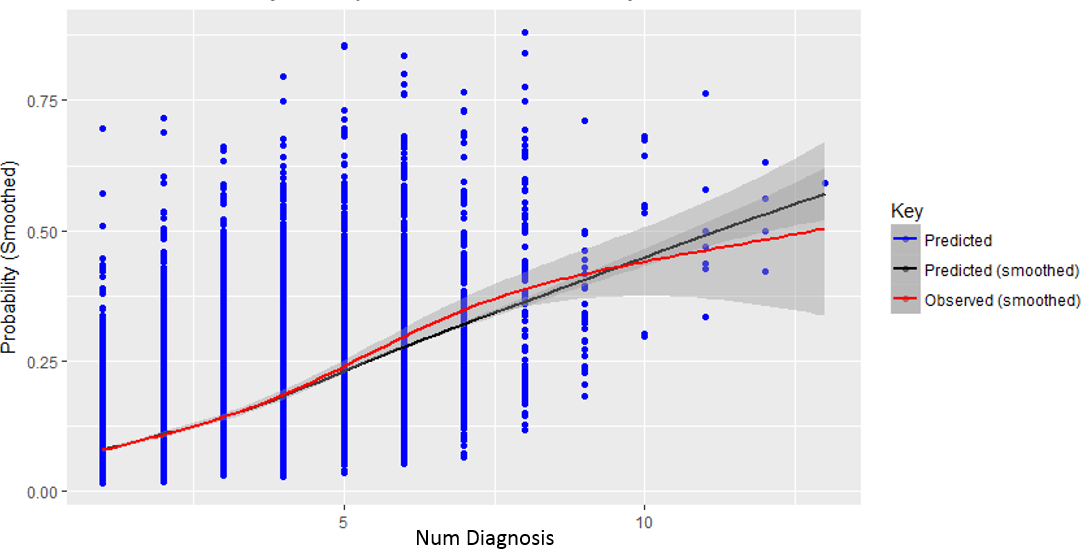


Supplementary Figure S4 – Predicted Probability of Hospitalisation within 1 Year by Number of Diagnoses (i.e. total number of distinct diagnosis families) for the Chronic-only subset of the Test dataset

Supplementary Data

Supplementary Table S7 shows predictor variables for 5 test cases while Supplementary Table S8 shows corresponding values for the linear predictor and predicted probability for each test case. For rows of Supplementary Table S7 corresponding to particular categories of a categorical variable, variables names are the concatenation of the predictor variable name and the specific category with no punctuation between them, in the style of R model output.

*Supplementary Table S7 - Specification of 5 Test Cases*

| **Variable (V_i_)** | **Coefficient (C_i_)** | **Test case**  **1** | **Test case**  **2** | **Test case 3** | **Test Case 4** | **Test Case**  **5** |
| --- | --- | --- | --- | --- | --- | --- |
| (Intercept) | -2.7551480 | | | | | |
| Age | -0.0379528 | 37 | 44 | 79 | 65 | 81 |
| Age_sq | 6.15945E-04 | 1369 | 1936 | 6241 | 4225 | 6561 |
| Age_cu | -1.04739E-06 | 50653 | 85184 | 493039 | 274625 | 531441 |
| Gender_fFemale | 0.2216384 | 0 | 1 | 0 | 0 | 1 |
| Ethnicity_fATSI | 0.4033629 | 0 | 0 | 1 | 0 | 1 |
| Ethnicity_fUnknown | -0.0662497 | 1 | 0 | 0 | 0 | 0 |
| BMI_7cat[25,30) | 0.0329420 | 1 | 0 | 0 | 0 | 0 |
| BMI_7cat[30,35) | 0.1939825 | 0 | 0 | 1 | 0 | 0 |
| BMI_7cat[35,40) | 0.2712273 | 0 | 0 | 0 | 0 | 0 |
| BMI_7cat40+ | 0.4643618 | 0 | 0 | 0 | 1 | 0 |
| BMI_7catNot recorded | 0.1395070 | 0 | 0 | 0 | 0 | 1 |
| SmokingStatus_4catex smoker | 0.1969316 | 0 | 0 | 0 | 1 | 0 |
| SmokingStatus_4catsmoker | 0.4057213 | 0 | 0 | 1 | 0 | 1 |
| SmokingStatus_4catUnknown | 0.1993470 | 0 | 0 | 0 | 0 | 0 |
| AnyAlcohol_3catDrinker | -0.2853232 | 1 | 0 | 0 | 0 | 0 |
| AnyAlcohol_3catNot recorded | -0.2301394 | 0 | 0 | 0 | 1 | 0 |
| Decile_IRSAD_f1 | -0.0123849 | 0 | 0 | 0 | 1 | 0 |
| Decile_IRSAD_f10 | -0.4622169 | 1 | 1 | 1 | 0 | 0 |
| Decile_IRSAD_f2 | -0.0742180 | 0 | 0 | 0 | 0 | 0 |
| Decile_IRSAD_f3 | 0.0044308 | 0 | 0 | 0 | 0 | 0 |
| Decile_IRSAD_f4 | -0.1857272 | 0 | 0 | 0 | 0 | 0 |
| Decile_IRSAD_f6 | -0.2340966 | 0 | 0 | 0 | 0 | 0 |
| Decile_IRSAD_f7 | -0.2468854 | 0 | 0 | 0 | 0 | 1 |
| Decile_IRSAD_f8 | -0.2990624 | 0 | 0 | 0 | 0 | 0 |
| Decile_IRSAD_f9 | -0.1834029 | 0 | 0 | 0 | 0 | 0 |
| Decile_IRSAD_fUnknown | -0.0301620 | 0 | 0 | 0 | 0 | 0 |
| meds.Statins.flag | -0.0152881 | 0 | 1 | 0 | 0 | 1 |
| meds.AntiCoagulants.flag | 0.2886091 | 0 | 0 | 0 | 0 | 0 |
| meds.AntiDepressants.flag | 0.2025163 | 0 | 0 | 0 | 0 | 1 |
| meds.AntiPsychotics.flag | 0.3923084 | 0 | 0 | 1 | 0 | 0 |
| meds.AntiInflammatory.flag | 0.1301034 | 1 | 1 | 1 | 0 | 0 |
| meds.Steroids.flag | 0.1488350 | 0 | 0 | 0 | 0 | 0 |
| NumDiseases | 0.3369661 | 1 | 2 | 6 | 2 | 9 |
| NumDiseases_sq | -0.0397663 | 1 | 4 | 36 | 4 | 81 |
| NumDiseases_cu | 0.0019304 | 1 | 8 | 216 | 8 | 729 |
| diseasegrp.respiratory.flag | -0.0715037 | 0 | 0 | 0 | 0 | 1 |
| diseasegrp.atrial_fibr.flag | 0.2234789 | 0 | 0 | 0 | 0 | 0 |
| diseasegrp.cardiovascular.flag | 0.4764327 | 0 | 0 | 1 | 0 | 1 |
| diseasegrp.osteoarthritis.flag | -0.2060183 | 0 | 0 | 0 | 0 | 1 |
| diseasegrp.osteoporosis.flag | 0.0595034 | 0 | 0 | 0 | 0 | 0 |
| diseasegrp.rheumatoid.flag | 0.1149149 | 0 | 0 | 0 | 0 | 0 |
| diseasegrp.mental_health.flag | 0.0686955 | 0 | 0 | 1 | 0 | 1 |
| diseasegrp.cancer.flag | 0.0600825 | 0 | 0 | 0 | 0 | 0 |
| diseasegrp.digestive.flag | 0.1796635 | 0 | 0 | 1 | 0 | 1 |
| diseasegrp.hypertension.flag | -0.1591489 | 0 | 1 | 0 | 1 | 0 |
| diseasegrp.bloodfats.flag | -0.3726723 | 1 | 1 | 0 | 0 | 1 |
| diseasegrp.chronic_kidney.flag | 0.0268266 | 0 | 0 | 0 | 0 | 0 |
| diseasegrp.diabetes_type_1.flag | 0.5844975 | 0 | 0 | 0 | 0 | 0 |
| diseasegrp.diabetes_type_2.flag | 0.1332004 | 0 | 0 | 1 | 1 | 0 |
| diseasegrp.venous_thrombo.flag | 0.3623621 | 0 | 0 | 0 | 0 | 1 |
| diseasegrp.other.flag | 0.5157983 | 0 | 0 | 1 | 0 | 0 |
| morb_v2.Hb_4catHigh | 0.4416708 | 0 | 0 | 0 | 0 | 0 |
| morb_v2.Hb_4catMed | 0.1546069 | 0 | 0 | 0 | 0 | 0 |
| morb_v2.Hb_4catNo test history | 0.0450222 | 1 | 0 | 1 | 0 | 0 |
| morb_v2.Platelets_4catHigh | 0.1398411 | 0 | 0 | 0 | 0 | 0 |
| morb_v2.Platelets_4catNo test history | -0.0147097 | 1 | 0 | 1 | 0 | 0 |
| morb_v2.ALT_4catHigh | -0.0619518 | 0 | 0 | 0 | 0 | 0 |
| morb_v2.ALT_4catMed | 0.0550267 | 0 | 0 | 0 | 0 | 0 |
| morb_v2.ALT_4catNo test history | -0.2940664 | 0 | 0 | 1 | 0 | 0 |
| morb_v2.GGT_4catHigh | 0.2209109 | 0 | 0 | 0 | 0 | 0 |
| morb_v2.GGT_4catMed | 0.1261477 | 0 | 0 | 0 | 0 | 0 |
| morb_v2.GGT_4catNo test history | 0.1979201 | 0 | 0 | 1 | 0 | 0 |
| morb_v2.HbA1c_4catHigh | 0.1746667 | 0 | 0 | 0 | 0 | 0 |
| morb_v2.HbA1c_4catMed | 0.1720256 | 0 | 0 | 0 | 0 | 0 |
| morb_v2.HbA1c_4catNo test history | -0.0461721 | 1 | 0 | 1 | 0 | 1 |
| morb_v2.Bilirubin_3catMed_or_High | 0.1803894 | 0 | 0 | 0 | 0 | 0 |
| morb_v2.Bilirubin_3catNo test history | 0.0784321 | 0 | 0 | 1 | 0 | 0 |
| morb_v2.Cholesterol_4catHigh | 0.0453054 | 0 | 0 | 0 | 0 | 0 |
| morb_v2.Cholesterol_4catMed | -0.0363534 | 1 | 1 | 0 | 0 | 0 |
| morb_v2.Cholesterol_4catNo test history | 0.1749709 | 0 | 0 | 1 | 0 | 0 |
| morb_v2.Creatinine_3catMed_or_High | 1.1349460 | 0 | 0 | 0 | 0 | 0 |
| morb_v2.Creatinine_3catNo test history | -0.1704331 | 1 | 0 | 1 | 0 | 0 |
| morb_v2.TAG_3catMed_or_High | 0.1234874 | 0 | 0 | 0 | 0 | 0 |
| morb_v2.TAG_3catNo test history | -0.0963018 | 0 | 0 | 1 | 0 | 0 |
| morb_v2.ACR_4catHigh | 0.3042750 | 0 | 0 | 0 | 0 | 0 |
| morb_v2.ACR_4catMed | 0.1151562 | 0 | 0 | 0 | 0 | 0 |
| morb_v2.ACR_4catNo test history | 0.1059512 | 1 | 1 | 1 | 0 | 1 |
| morb_v2.LDL_4catHigh | -0.0585925 | 1 | 1 | 0 | 0 | 0 |
| morb_v2.LDL_4catMed | -0.0539600 | 0 | 0 | 0 | 0 | 0 |
| morb_v2.LDL_4catNo test history | 0.1556545 | 0 | 0 | 1 | 0 | 0 |
| morb_v2.eGFR_4catHigh | 0.0891486 | 0 | 0 | 0 | 0 | 0 |
| morb_v2.eGFR_4catMed | 0.0950474 | 0 | 0 | 0 | 0 | 0 |
| morb_v2.eGFR_4catNo test history | 0.0459790 | 1 | 0 | 1 | 0 | 0 |
| morb_v2.BP_4catHigh | 0.2946162 | 0 | 0 | 0 | 0 | 0 |
| morb_v2.BP_4catMed | 0.1839175 | 0 | 0 | 0 | 0 | 0 |
| morb_v2.BP_4catNo test history | 0.0112105 | 0 | 0 | 0 | 0 | 0 |
| Gender_fFemale:diseasegrp.  cardiovascular.flag | -0.2108839 | 0 | 0 | 0 | 0 | 1 |
| Gender_fFemale:diseasegrp.  respiratory.flag | 0.0570566 | 0 | 0 | 0 | 0 | 1 |
| Gender_fFemale:diseasegrp.  diabetes_type_1.flag | 0.2492323 | 0 | 0 | 0 | 0 | 0 |
| Gender_fFemale:diseasegrp.other.flag | -0.1721620 | 0 | 0 | 0 | 0 | 0 |
| Gender_fFemale:diseasegrp.  osteoporosis.flag | -0.2475655 | 0 | 0 | 0 | 0 | 0 |
| Gender_fFemale:diseasegrp.  chronic_kidney.flag | -0.1122160 | 0 | 0 | 0 | 0 | 0 |
| Gender_fFemale:diseasegrp.  mental_health.flag | -0.0643675 | 0 | 0 | 0 | 0 | 1 |
| Gender_fFemale:diseasegrp.  hypertension.flag | -0.0890935 | 0 | 1 | 0 | 0 | 0 |
| Gender_fFemale:diseasegrp.  diabetes_type_2.flag | -0.0002784 | 0 | 0 | 0 | 0 | 0 |

*Supplementary Table S8 - Predicted Probability and Risk Group for 5 Test Cases*

| **Outcome** | **Test case**  **1:**  **low predicted probability** | **Test case**  **2:**  **low predicted probability** | **Test case**  **3:**  **high predicted probability** | **Test Case**  **4:**  **variety** | **Test Case**  **5:**  **many co-morbidities** |
| --- | --- | --- | --- | --- | --- |
| linear predictor | -4.222820 | -3.527184 | 1.200765 | 1.984220 | 0.263264 |
| predicted probability | 0.014446 | 0.028549 | 0.768661 | 0.120870 | 0.565439 |
| Risk Group | Bottom 20% | Bottom 20% | Top 1% | Top 30% | Top 1% |
